# Health Literacy and Self‐Efficacy in Parents of Toddlers—A Cross‐Sectional Study

**DOI:** 10.1002/nop2.70078

**Published:** 2025-02-17

**Authors:** Ragnhild Makhotso Egenberg Søreide, Marie Hamilton Larsen, Astrid Klopstad Wahl, Merete Kristin Tschamper, Kristin Hjorthaug Urstad

**Affiliations:** ^1^ Community of Stavanger Stavanger Norway; ^2^ Lovisenberg Diaconal University College University of Oslo Oslo Norway; ^3^ University of Oslo, Oslo University Hospital Oslo Norway; ^4^ University of Oslo Oslo Norway; ^5^ University of Stavanger Stavanger Norway; ^6^ VID Specialized University Oslo Norway

**Keywords:** child, health literacy, health literacy questionnaire–parent version (HLQ–p), parent, self‐efficacy

## Abstract

**Aim:**

Young children depend on their parents to develop in a positive and healthy way. For parents to promote and maintain good health for their children, parent's level of health literacy and self‐efficacy is important. The aim of this study was to explore health literacy in parents of toddlers and investigated the associations between parental health literacy and self‐efficacy.

**Design:**

The study utilised a quantitative cross‐sectional design.

**Method:**

Parents (*N* = 76) with children between the ages of 6 weeks and 12 months were recruited from a public health centre in Norway. Multidimensional health literacy was measured using the Health Literacy Questionnaire–Parent version while self‐efficacy was measured using the General Self‐Efficacy Scale. The health literacy domains are presented by descriptive analysis. The associations among health literacy domains, demographic variables and self‐efficacy were analysed using bivariate correlations and non‐parametric tests.

**Results:**

Health literacy scores were overall high, and self‐efficacy was positively associated with all nine health literacy dimensions. The highest score of health literacy was found for the *feel that healthcare providers understand and support my child's situation* dimension (median 3.8 with interquartile range 3.3–4.0). The lowest scores of health literacy were found for the *appraisal of health information* dimension (median 3.2 with interquartile range 3.0–3.8). Self‐efficacy had a score of median 3.5 with interquartile range 3.2–3.7.

**Conclusion:**

The parents of toddlers in this study had high parental health literacy and self‐efficacy. Self‐efficacy was positively associated with all nine health literacy constructs. Future research should include multiple measure points to assess the dynamic variation on both parental health literacy, self‐efficacy and demographic variables.

**Patient or Public Contribution:**

No patient or public contribution.

## 1 Background

Health literacy plays an essential role in people's health decisions and behaviours (Nutbeam and Muscat 2020; Steunenberg et al. 2018); consequently, parents' health literacy is essential for a child's development (DeWalt and Hink 2009; Jarosz and Gugushvili 2020; Keim‐Malpass, Letzkus, and Kennedy 2015). Health literacy represents personal knowledge and skills to access, understand, appraise and use health information to promote and maintain good health for themselves and those around them, and people are dependent on the organisational systems to succeed (Nutbeam and Muscat 2021).

Many studies around the world have investigated parental health literacy in a public health context with children in school age. For instance, a recent study conducted in Germany investigated health literacy in 4217 parents with children in the age between 6 and 10 years (de Buhr and Tannen 2020). The study demonstrated negative associations between low parental health literacy and many essential aspects likely to negatively impact their child health such as less healthy nutrition, less exercise and worse toothbrushing habits (de Buhr and Tannen 2020). Another large study of 5877 Californian immigrants with children in the same age group reported positive associations between adequate parental health literacy and their child's overall physical health (Lee et al. 2018). Furthermore, similar associations have been demonstrated across time. In a study collecting data from 34 countries in Europe and Asia, positive associations were demonstrated between sufficient parental health literacy and their children's adult body height (Jarosz and Gugushvili 2020).

Nurses work with health promotion and disease prevention, and a crucial part of this work is supporting, guiding and educating parents to strengthen their parental health literacy and self‐efficacy. Being parents to a baby can be stressful and challenging, and most parents need help or guidance from time to time. In Norway, all children and their parents get legally required and free‐of‐charge follow‐up from birth until they start school. This follow‐up is mainly done by the public health nurses and doctors at the public health centres (Helsedirektoratet 2022). The first ordinary consultation is a home visit, and the first consultation conducted at the public health centre is when the child is 6 weeks old. Most of the ordinary consultations in the infant healthcare programme are during the child's first year. Here, the child gets health checkups, vaccines and parents can address their questions and concerns. The public health nurse will guide parents in reflecting about their parenting role, child development challenges concerning interaction with the healthcare system, guide them on where to find reliable health information and how they can get support from others when necessary. This particular governmental health service aims to help parents experience mastery in their parental role and plays an essential role in following up on children's health and development and strengthening parents' health literacy (Helsedirektoratet 2022).

To offer parents high‐quality follow‐up, it seems vital to understand their parental health literacy strengths and challenges, including how they access, understand, appraise and use information and services to promote and maintain good health and well‐being for their child (Nutbeam and Muscat 2021). Although many studies around the world have investigated parental health literacy in a public health context, the majority of health literacy research has used instruments measuring reading and numerical skills, known as functional health literacy (DeWalt and Hink 2009; Rademakers and Heijmans 2018). Young children are especially dependent on their parents' skills and ability to conduct tasks related to understanding and critically assessing health information, to navigate the healthcare system, and to achieve successful collaboration with healthcare providers (Bánfai‐Csonka et al. 2022). To achieve a broader and more in‐depth knowledge about these tasks and skills, parental health literacy needs to be evaluated by comprehensive instruments that incorporate the full multidimensional nature of health literacy. According to Nutbeam and Muscat (2020, 117), ‘a person with a high level of observable health literacy skills may experience real challenges in applying them in an environment (like a hospital) or in interacting with a person (like a physician) that they find unfamiliar and intimidating’. Furthermore, critical health literacy requires the highest levels of skills to critically analyse and use information to exert greater control over life events and situations to appraise information of relevance to health (Sykes et al. 2013). The Health Literacy Questionnaire (HLQ) (Osborne et al. 2013) includes all three levels of health literacy (i.e., functional, interactive and critical health literacy). Assessing parental health literacy using the HLQ can help get more information about parents' strengths and challenges, thereby facilitating public health services for families.

Self‐efficacy is closely linked to health literacy (Baker 2006; Jafari, Tavakoly Sany, and Peyman 2021; Keim‐Malpass, Letzkus, and Kennedy 2015; Nutbeam 2015). Self‐efficacy refers to ‘beliefs in one's capabilities to organize and execute the courses of action required to produce given attainments’ (Bandura 1997, 3). Thus, self‐efficacy beliefs influence how people think, feel, motivate themselves and act (Bandura 2013). Parents' self‐efficacy therefore plays an important role in the health and well‐being of children (Albanese, Russo, and Geller 2019) and higher parental health literacy has shown to have positive associations with higher parental self‐efficacy (Tan and Karakas 2022). The recent study of Tan and Karakas (2022) focusing on parents with children with a chronic disease found associations between total scores of health literacy and self‐efficacy, measured by the General Self‐efficacy Scale. A systematic review focusing on associations between health literacy and self‐efficacy in the context of diabetic patients support these findings (Xu, Leung, and Chau 2018). However, the review states the importance of a multidimensional approach. Clear associations were found regarding communicative and critical health literacy, while the association was less convincing concerning a functional health literacy (Xu, Leung, and Chau 2018). This emphasises the importance of critical consideration in use of measurement tools when investigating possible relationships between self‐efficacy and health literacy.

The rationale for the study to be conducted among the parents of toddlers in Norway on health literacy and self‐efficacy is that many studies investigate health literacy and self‐efficacy in parents with higher risk of low health literacy, parents that have children in school age or parents that have children with specific medical illnesses. To the best of our knowledge, parental health literacy has not been assessed using the HLQ–p in a public health context in Norway. Therefore, this study aimed to explore health literacy among parents of toddlers (6 weeks–12 months old) from a multidimensional perspective by utilising the HLQ–p and to explore the HLQ–p domains' associations with self‐efficacy and sociodemographic factors. This knowledge might strengthen nurses and other health workers competence in future work with families.

## 2 Methods

### 2.1 Study Design

This study adopted a cross‐sectional design and included parents of children aged 6 weeks up to 12 months. It was conducted in accordance with the STROBE (Strengthening the Reporting of Observational Studies in Epidemiology) guidelines for the reporting of observational studies (von Elm et al. 2007).

### 2.2 Study Context and Data Collection

This study took place over 4 weeks in November and December 2021 in a public health centre in Norway where parents attend for legally required consultations in the infant healthcare program during the child's first year. Participant recruitment was done via convenience sampling. Only parents who had a consultation with their child at the public health centre during the time of the study were invited to participate. It was the public health nurses that invited parents to participate at the end of their consultation. Inclusion criteria were as follows: being over 18 years old and the parent of a child between 6 weeks and 12 months of age and being able to read and understand Norwegian. Parents that did not meet the inclusion criteria were excluded from participating. Parents who agreed to participate received a written informed consent form and the paper‐based questionnaire from the public health nurse and completed the forms immediately after the consultation. The average duration for participants to complete the survey was 10–15 min. After completing the questionnaire and signing the consent form, the participants delivered the forms in a secured collection box at the public health centre. Only the authors RES and KHU had access to the completed questionnaires. Parents that declined to participate were not asked about their reasons, and we do not know if they differ from the participants in any way.

### 2.3 Measures

The questionnaire comprised three sections: sociodemographic and health variables, the General Self‐Efficacy Scale (GSE; Røysamb, Schwarzer, and Jerusalem 1998) and the Health Literacy Questionnaire–Parent version (HLQ–p; Wahl et al. 2022). Both HLQ‐p and GSE are already validated, and the data from this study were validated by using Cronbach's alfa; the specific results are shown in Table 2.

### 2.4 Sociodemographic and Health Variables

**FIGURE 1:**
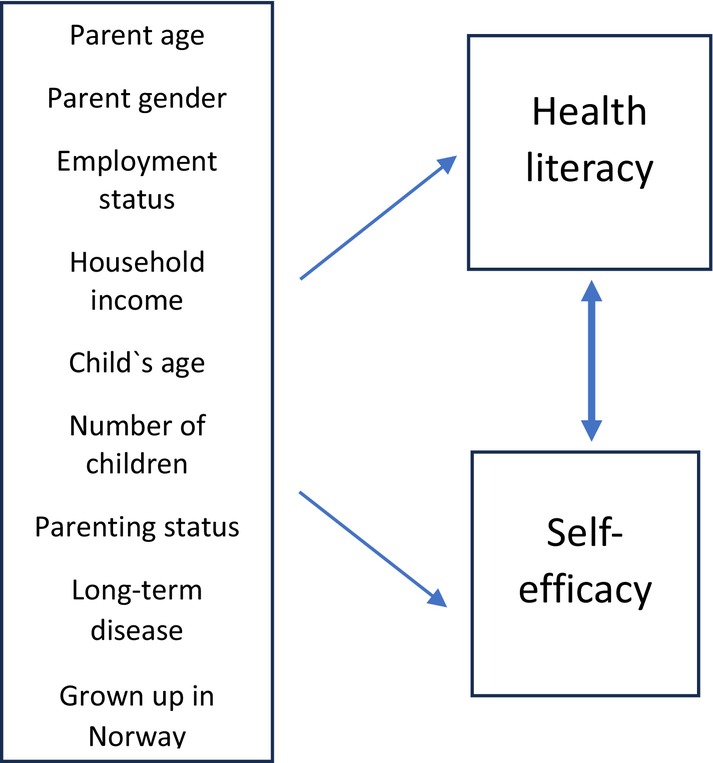
Overview over study variables.

The selection of variables was based on ‘The health literacy model proposed’ by Sørensen et al. (2012). The model acknowledge that health literacy is influenced by varies personal and social determinants (Sørensen et al. 2012). The questionnaire included questions about the parent's gender, age, educational level, employment status, household income, child's age, number of children, parenting status (i.e., co‐parenting, living together with the other parent), whether the parent spent their childhood in Norway and the history of any long‐term diseases for both the parent and the child. All the questions had pre‐categorised response options to secure anonymity. See Figure 1 for an overview over study variables.

### 2.5 Health Literacy Questionnaire–Parent Version (HLQ–p)

Health literacy was measured using the Health Literacy Questionnaire–Parent version (HLQ–p), which originated from the Health Literacy Questionnaire (HLQ). The HLQ is widely used and has been translated into more than 30 languages (Rademakers et al. 2020). The HLQ has also been validated in different Norwegian contexts (Urstad et al. 2022; Wahl et al. 2022). The generic HLQ was adapted to a parental context and translated in collaboration with the original developers of the instrument (Hawkins, Elsworth, and Osborne 2018; Wahl et al. 2022). The HLQ‐p has been validated in a parental context with adaption processes and CFA analysis that supported the relevance, understanding and theoretical structure of the instrument (Wahl et al. 2022). Cronbach's alfa was 0.70–0.87 in all nine HLQ scales, and cognitive interviews indicated that parents interpreted the HLQ‐p items as intended (Wahl et al. 2022).

The HLQ–p is multidimensional and comprises 44 items within nine domains, each domain representing a specific construct: (1) Feel that healthcare providers understand and support my child's situation, (2) Having sufficient information to manage my child's health, (3) Actively managing my child's health, (4) Experience social support for my child's health, (5) Appraisal of health information, (6) Ability to actively engage with healthcare providers, (7) Navigating the healthcare system, (8) Ability to find good health information and (9) Understand health information well enough to know what to do (Osborne et al. 2013; Wahl et al. 2022).

In the first five domains, items are scored from 1 to 4 (strongly disagree, disagree, agree, strongly agree); in the four last domains, the items are scored from 1 to 5 (cannot do or always difficult, usually difficult, sometimes difficult, usually easy, always easy) (Hawkins, Elsworth, and Osborne 2018; Osborne et al. 2013; Wahl et al. 2022). There are no cut‐off scores, but a higher score indicates a higher level of health literacy (Osborne et al. 2013).

### 2.6 General Self‐Efficacy Scale (GSE)

The GSE contains 10 statements reflecting an individual's belief in their ability to respond to novel or difficult situations (Schwarzer and Jerusalem 1995). Each statement includes a 4‐point response scale, ranging from ‘not at all true’ to ‘completely true’. The GSE score range from 10 to 40 points, where a higher score indicates better self‐efficacy. The questionnaire has been translated into Norwegian, and it shows satisfactory reliability and validity (Røysamb, Schwarzer, and Jerusalem 1998). As the GSE was not explicitly written for parents, these questions were introduced with a short text for contextualisation.

### 2.7 Statistical Analyses

Statistical analyses were performed using SPSS software version 25/26. In instances where respondents did not provide responses to all items within a particular scale of the HLQ, the scale was excluded from the analysis. Descriptive analyses of the nine different HLQ–p domains and the GSE scale, including median, variance, quartiles and Cronbach's alpha, were performed. Median as measurement for central tendency is recommended when the study sample is limited (Johannessen, Tufte, and Christoffersen 2016). The interquartile range, indicating the score of the middle 50% of the sample, is presented together with minimum and maximum scores due to skewed data in the sample. Due to the limited number of respondents, skewed data sample and the rough categorisation of sociodemographic variables, only robust non‐parametric analyses were performed. When analysing limited and skewed data with non‐parametric tests, Spearman's rho is recommended to assess the correlation between the different health literacy domains and GSE (Polit and Beck 2017).

Some categories had a low response rate and were merged with the closest category to strengthen the results of the analyses (Johannessen, Tufte, and Christoffersen 2016). For example, ‘number of children’, which originally had the categories one, two and three or more, was merged into two new variables: ‘one child’ and ‘two or more children’. Becoming a parent for the first time can be extra stressful, and this merging could be clinically meaningful (Pedersen, Ballo, and Nilsen 2019).

The Mann–Whitney U test and Kruskal–Wallis test were used to examine the association between all the health literacy domains and the different sociodemographic variables. As the current study included multiple variables, there is risk a of type 1 error. Because of this, the Bonferroni correction was used (Polit and Beck 2017). The correction was done by dividing the original alpha level (0.05) by the number of tests being performed resulting in an adjusted *p*‐value of 0.004.

### 2.8 Ethical Considerations

The study was approved by the Norwegian Center for Research Data (REDACTED). The study protocol was also reviewed by the Regional Committee for Medical and Health Research Ethics in Norway (REK), which stated that, in according with Norwegian law, the study could be carried out without their approval (REDACTED). This study was conducted according to the Declaration of Helsinki. All participants were given written information and they signed a consent form. The participants were informed about the confidential handling of information and that they could withdraw their consent at any time without explanation. It was also emphasised that participation and scoring would not affect the routine follow‐up care from the public health centre.

## 3 Results

### 3.1 Sample Characteristics

Out of 95 parents who were asked to participate in the study, 76 agreed (80% response rate). The majority of the respondents had a high level of education, were employed, earned a high income, shared parental responsibility and had spent their childhood in Norway; in addition, most parents and children were without chronic illnesses. See descriptive demographics for study participants in Table 1.

**TABLE 1:** Descriptive demographics adjusted before the analyses (Total *N* = 76).

	*N*	*n* (%)
Gender, female	74	58 (78)
Age of parent, years	74	
≤ 32		27 (37)
≥ 33		47 (63)
Education level	74	
Primary, secondary, high school, or less than 4 years of university/college		30 (40)
University/college (4 years or more)		44 (60)
Work status, working	75	71 (95)
Household income, NOK^a^	75	
≤ 599,0.000		14 (19)
600,000–899,999		15 (20)
≥ 900,000		46 (61)
Age of child, months	74	
1–3		23 (31)
4–6		36 (49)
7–12		15 (20)
Number of children	74	
One child		43 (58)
Two or more children		31 (42)
Co‐parenting, shared	74	71 (96)
Living together with the other parent	73	71 (97)
Parent spent childhood in Norway	75	66 (88)
No long‐term disease in parent	74	72 (97)
No long‐term disease in child	75	68 (91)

^a^ NOK = Norwegian kroner (as of 20 May 2022, the exchange rate was 1000 NOK ≈97.36 € ≈US$103.17).

### 3.2 Health Literacy Scores

All nine health literacy domains had a high median score, ranging from 3.2 to 3.8 in part 1 (response categories 1–4) and 4.1 to 4.4 in part 2 (response categories 1–5). The domain with the highest median was domain 1 (*feel that healthcare providers understand and support my child's situation*; response categories 1–4), with a median of 3.8, interquartile range Q_1_–Q_3_: 3.3–4.0 (α 0.69). The domain with the lowest median was domain 5 (i.e., *appraisal of health information*; response categories 1–4), with a median of 3.2, Q_1_–Q_3_: 3.0–3.8 (α 0.65). All the respondents that had low score in one domain, scored high in other domains. In all health literacy domains except domain 7 (*navigating the healthcare system*), at least 75% of the respondents scored between the two highest response categories. Domain 7 had a first quartile value of 3.8 (response categories 1–5). See Table 2 for more details.

**TABLE 2:** Descriptive statistics and reliability testing of Health Literacy Questionnaire–Parent version (HLQ–p) and General Perceived Self‐Efficacy Scale (GSE) (Total *N* = 76).

Health literacy scores (HLQ–p)^a^	*n* (%)	Median	Q_1_–Q_3_	Min–max	*α*
1. Feel that healthcare providers understand and support my child's situation (1–4)	73 (96)	3.8	3.3–4.0	2.5–4	0.69
2. Having sufficient information to manage my child's health (1–4)	73 (96)	3.5	3.3–4.0	2.5–4	0.73
3. Actively managing my child's health (1–4)	73 (96)	3.6	3.3–3.8	2.4–4	0.69
4. Experience social support for my child's health (1–4)	73 (96)	3.6	3.2–3.8	2.2–4	0.82
5. Appraisal of health information (1–4)	73 (96)	3.2	3.0–3.8	2.2–4	0.65
6. Ability to actively engage with healthcare providers (1–5)	76 (100)	4.2	4.0–4.8	3.2–5	0.81
7. Navigating the healthcare system (1–5)	74 (97)	4.1	3.8–4.5	2.8–5	0.82
8. Ability to find good health information (1–5)	74 (97)	4.2	4.0–4.8	3.0–5	0.84
9. Understand health information well enough to know what to do (1–5)	76 (100)	4.4	4.0–4.8	3.2–5	0.77
Perceived self‐efficacy (GSE) (1–4)	75 (99)	3.5	3.2–3.7	2.4–4	0.82

Abbreviations: Q_1_: first quartile; Q_3_: third quartile; α: Cronbach's alpha.

^a^ Range of possible values.

### 3.3 Associations Between Health Literacy and Demographics

Three demographic variables had a statistically significant association with a health literacy domain: respondents who were 32 years or younger scored higher on domain 2 (*having sufficient information to manage my child's health*; *p* = 0.002) than those of 33 years or older. 7.5% of the variation could be explained by the age. Domain 2 was also associated with parents' education level (*p* = 0.012) as respondents with primary, secondary, high school or less than 4 years of university/college scored higher than respondents with 4 years or more of university/high school. Education level could explain 9.0% of the variation. The third association was between domain 1 (*feel that healthcare providers understand and support my child's situation*) and age of the child (*p* = 0.004). The assumptions for the Kruskal–Wallis test were not satisfied, so further analyses were not conducted.

None of the results on associations between the health literacy scores and demographics were significant after the Bonferroni correction of multiple tests was applied. Appendix 1 displays more information about the results from the tests of associations between health literacy and demographics.

### 3.4 Association Between Health Literacy and Self‐Efficacy

Self‐efficacy (with response categories 1–4) had a median score of 3.5, Q_1_–Q_3_: 3.2–3.7 (α 0.82). All nine health literacy domains were significantly correlated with self‐efficacy (*r*_*s*_ = 0.24 to 0.40; see Table 3). Higher self‐efficacy correlated with higher health literacy. The strongest correlation was between self‐efficacy and domain 9 (*understand health information well enough to know what to do*; *r*_*s*_ = 0.40, *p* < 0.001). Self‐efficacy also had a medium‐strong correlation with domain 6 (*ability to actively engage with healthcare providers*; *r*_*s*_ = 0.37, *p* = 0.001) and domain 4 (*experience social support for my child's health*; *r*_*s*_ = 0.34, *p* = 0.004). The lowest correlation was between self‐efficacy and domain 1 (*feel that healthcare providers understand and support my child's situation*; *r*_*s*_ = 0.24, *p* = 0.046) (See Table 3).

**TABLE 3:** Correlation between health literacy scales and self‐efficacy (Total *N* = 76).

Health literacy scale	*n*	Self‐efficacy	
		*r*_*s*_	*p*
1. Feel that healthcare providers understand and support my child's situation	73	0.24	0.046^a^
2. Having sufficient information to manage my child's health	73	0.31	0.008^b^
3. Actively managing my child's health	73	0.28	0.016^a^
4. Experience social support for my child's health	73	0.34	0.004^b^
5. Appraisal of health information	73	0.24	0.039^a^
6. Ability to actively engage with healthcare providers	75	0.37	0.001^b^
7. Navigating the healthcare system	73	0.28	0.018^a^
8. Ability to find good health information	74	0.27	0.022^a^
9. Understand health information well enough to know what to do	75	0.40	< 0.001^c^

Abbreviations: *p*: sig (2‐tailed); *r*_*s*_, Spearman's rho.

^a^ Significant at the 0.05 level.

^b^ Significant at the 0.01 level.

^c^ Significant at the 0.001 level.

## 4 Discussion

This study aimed to explore health literacy strengths and challenges in parents of toddlers and to investigate the association between parents' health literacy and self‐efficacy. The developers of HLQ have not provided any cut‐off values for high/ low health literacy, as health literacy is a complex concept with multiple dimension (Osborne et al. 2013). They claim that even if a cut‐off approach allows for ease of use, there is limited scientific evidence to determine if a particular level of health literacy puts a person at risk of a poor health event (Osborne et al. 2022). While individuals may encounter difficulties in certain aspects, such as comprehending health information independently, they may also possess strengths, such as support from clinicians or family and knowledge of accessing healthcare services. This renders the notion of high or low overall health literacy as not appropriate. However, since the parents in our sample generally scored in the upper range of the HLQ scoring options, related to the maximum levels of four (domains 1 to 5) and five (domains 6 to 9), we have chosen to describe this as high health literacy.

The respondents in this study had a high score in domain 4 (*experience social support for my child's health*). Some of the positive results of demographic variables in this study; working, living together with the other parent, and neither child nor parent have long‐term diseases, might increase the possibility of experiencing social support for their child's health. These findings correspond with Nutbeam's description of health literacy as an asset, where social determinants for health are crucial for health literacy (Nutbeam 2008). High score in this domain is in contrast with the study measuring HLQ–p in parents of children with epilepsy, where the score was distinctly lower (Tschamper et al. 2022). In recent years, social support has been emphasised as an essential aspect when working towards a strengthened public health system (Barstad and Sandvik 2015; Helsedirektoratet 2018c). Assessing social support is quite new in studies concerning health literacy, and according to Muscat et al. (2022), that is a particular strength of the HLQ, which the HLQ–p is based on.

An interesting observation in the current study was that parents of younger age (32 years or younger) seemed to be particularly well informed as they had higher scores on domain 2 (*having sufficient information to manage my child's health*). The previous mentioned cross‐sectional study by de Buhr and Tannen (2020) also aimed to understand parental health literacy utilising children in elementary and secondary schools in Germany. The study assesses the implications for children's health by using the European Health Literacy Survey questionnaire (HLS‐EU‐16). Two major determinants of high parental health literacy in that study were high socio‐economic status and older parental age, which is in contrast with our age findings (de Buhr and Tannen 2020). One might wonder if this result in our study is related only to the small sample size or that there might be other underlying explanations not assessed in the study. The majority of the parents and their children in this study did not have long‐term illnesses. Thus, general health information might be sufficient to manage their child's health in many situations. Furthermore, our findings might also partly be explained by potentially new patterns in the younger generation's health information seeking. The rapid proliferation of and access to health information on the Internet have created new possibilities related to finding health information. People are increasingly comfortable using the Internet as a prime source of basic health information and sharing experiences through social network platforms and parent forums (Colditz, Woods, and Primack 2018). This might also explain the general high score in domain 8 (*ability to find good health information*).

Another interesting observation in this study is the fact that highly educated parents scored lower on domain 2 (*having sufficient information to manage my child's health*) than parents with less than 4 years of higher education. This is in contrast with general findings of other health literacy studies where higher education is considered a strength. (Sudhakar et al. 2020; Jansen et al. 2018). Our clinical experience confirms that being parents to a toddler can be stressful and challenging, and regardless of education, parents need reliable health information and social support. Children do not come with a user manual. This underpins the need for social support. Despite high academic education level among parents, it seems as if it still ‘takes a village to raise a child’.

How the child's age was associated with domain 1 (*feel that healthcare providers understand and support my child's situation*), is also worth mentioning. When the child is 4–6 months, the family has had close follow‐up by the same public health nurse for some time. This period enables a trusting relationship between parents and the public health nurse. The child's development is assessed closely, and continuity is of vital importance (Enlow, Passarella, and Lorch 2017).

There isn't a specific ‘cut‐off’ score in the GSE as far as we have been able to explore; instead, interpretation may depend on the context of the study. As self‐efficacy scores were high in the current study, our results indicate that parents had high beliefs in their ability to handle different situations regarding their child's health. Higher self‐efficacy was further significantly associated with higher health literacy. This finding is in line with results from previous studies (Dahl et al. 2020; Fong et al. 2018; Jafari, Tavakoly Sany, and Peyman 2021; Keim‐Malpass, Letzkus, and Kennedy 2015; Lee et al. 2018). One might argue that the positive correlation between health literacy and self‐efficacy is not surprising; hence, they are both measured by self‐reported information. On the other hand, a recent study conducted by Medina et al. (2022) did not find any significant correlation between health literacy and self‐efficacy measured in patients with diabetes. In the study of Medina et al. (2022), the instrument measuring health literacy was one‐dimensional, measuring functional health literacy only. In the current study, the health literacy instrument was multidimensional. It seems important to investigate correlations between self‐efficacy and health literacy utilising a multidimensional approach to get insight in which aspects of health literacy that might be particularly linked to parents' beliefs in own ability to cope with the health of their child. Our results of higher self‐efficacy can be seen in relationship with the demographic variables ‘working’ and having a younger age (below 40 years). This is in line with a Norwegian study, where ‘male gender’ and ‘being employed’ were related to higher self‐efficacy, especially among people of younger age (Bonsaksen et al. 2019). This can also be related to the findings from Albanese, Russo, and Geller (2019), where parental self‐efficacy might have a relation to demographic variables.

The HLQ–p does not define the concept of health and therefore the responders´ perceptions of health can be comprehended differently. This variation might be explained by respondents' everyday life and the context in which health is discussed (Mæland 2016). When asking parents with a healthy child about health, they might associate health with nutrition, physical activity and general development. Meanwhile, parents of a child with a chronic illness might primarily associate health with their child's illness. In domain 8 (*ability to find good health information*), parents of children with epilepsy scored much lower than parents in this current study (Tschamper et al. 2022). This emphasises the importance of parents' context when analysing the HLQ–p results.

An explanatory factor for the overall high levels of health literacy in the current study might be that the respondents were a relatively homogeneous group, with a high socio‐economic status, in terms of education, work, income and childhood in Norway. This is in line with other studies describing that socio‐economic status is strongly associated with parents' high health literacy (Zaidman et al. 2023; de Buhr and Tannen 2020). Parents who had difficulties understanding Norwegian were excluded from this study as the questionnaires were only offered in Norwegian and no interpreter services were available. If we had included multiple public health centres in different parts of the community or provided multi‐language questionnaires, our study might have included a more representative study population. However, as this is the first study in a public health context measuring health literacy utilising the multidimensional HLQ–p, it might indicate some trends in demographic differences that could be interesting for further investigations.

The HLQ and GSE are both self‐reported measures. It is well‐known that self‐reported data do not always correlate with actual skill‐levels (Dang, King, and Inzlicht 2020). This could be a potentially bias that needs awareness when interpreting the results, especially since the results are near ceiling effect. However, both health literacy and self‐efficacy have been assessed in numerous surveys in different contexts and cannot be measured by objective instruments only (Urstad et al. 2022; Larsen et al. 2022). Consequently, the scientific value of the self‐reported data should be considered valuable.

The current study measured health literacy and self‐efficacy at one point in time. As health literacy and self‐efficacy changes through life (Sørensen et al. 2012), multiple measure points would increase our insight of patterns regarding parents´ ability to promote good health for their children. Multiple measure points for the same participants could also give insight on how health literacy and self‐efficacy changes depending on sosio‐economic status or when parents face different challenges, whether it is sick children, parenting challenges or particularly difficult times.

The study's findings suggest important implications for practice, highlighting the dynamic nature of health literacy and self‐efficacy. Parents require social support to navigate their daily lives and the challenges they encounter. Consistent follow‐up by the same healthcare professionals is recommended. Additionally, it is essential for nurses to engage in discussions with parents about how they seek information and what specific details they need, to ensure that health information is more accessible and relevant to them.

### 4.1 Study Limitations

A higher risk of a type I error is possible with multiple testing, and a true null hypothesis might be rejected. Thus, a Bonferroni correction was applied, with the consequence that none of the results were significant anymore. This, on the other hand, increases the risk of type II error, where a false null hypothesis is accepted (Polit and Beck 2017). In the results, we present the initial figures that were statistically significant. We recognise that these results are statistically weak, and any interpretations should be done with caution.

A sample size determination was not calculated for in the current study. In comparison with other HLQ studies with similar study design (Urstad et al. 2022), the responders in some of the categories in our study are somewhat low. The rough categorisation was made to de‐identify the respondents, but this approach also made it more difficult to appraise the clinical significance of the results as none of the results were significant after the Bonferroni correction of multiple tests was applied. We acknowledge that a larger sample size would have provided increased statistical power (Julious 2023); however, the current sample size was constrained by practical considerations. Despite this limitation, the findings from our study still offer valuable exploratory insights and can serve as a basis for further investigation into this area.

## 5 Conclusion

This is the first study investigating multidimensional parental health literacy in a public health context in Norway. The parents of toddlers in this study had high parental health literacy and self‐efficacy. Self‐efficacy was positively associated with all nine health literacy constructs. As health literacy and self‐efficacy changes through life, multiple measure points would increase our insight of patterns regarding parents´ ability to promote good health for their children. How demographic variables associate with the dynamic parental health literacy and self‐efficacy, would be interesting to explore in future research.

## Author Contributions

Substantial contribution to conception and design: R.M.E.S., M.H.L., A.K.W., M.K.T., K.H.U. Substantial contribution to acquisition of data: R.M.E.S., K.H.U. Substantial contribution to analysis and interpretation of data: R.M.E.S., M.H.L., A.K.W., M.K.T., K.H.U. Drafting the article; R.M.E.S., K.H.U. Critically revising the article for important intellectual content: R.M.E.S., M.H.L., A.K.W., M.K.T., K.H.U. Final approval of the version to be published: R.M.E.S., M.H.L., A.K.W., M.K.T., K.H.U.

## Ethics Statement

The study was approved by the Norwegian Center for Research Data (REDACTED). The study protocol was also reviewed by the Regional Committee for Medical and Health Research Ethics in Norway (REK), which stated that, in according with Norwegian law, the study could be carried out without their approval (REDACTED). This study was conducted according to the Declaration of Helsinki.

## Consent

All participants were given written information and a signed informed consent form. The participants were informed about the confidential handling of information and that they could withdraw their consent at any time without explanation.

## Conflicts of Interest

The authors declare no conflicts of interest.

## Data Availability

The data supporting this study's findings are available from the corresponding author upon reasonable request.

## Appendix 1

### Association between health literacy scales and demographic variables

**Table:**

	1. Feel that healthcare providers understand and support my child's situation	2. Having sufficient information to manage my child's health	3. Actively managing my child's health	4. Experience social support for my child's health	5. Appraisal of health information
	*p*	*p*	*p*	*p*	*p*
Gender^a^	0.661	0.269	0.297	0.852	0.253
Age of parent^a^	0.147	**0.022***	0.219	0.142	0.342
Education level^a^	0.402	**0.012***	0.055	0.081	0.740
Employment status^a^	0.199	0.392	0.194	0.788	0.910
Household income^b^	0.449	0.352	0.952	0.274	0.417
Age of child^b^	**0.004***	0.440	0.207	0.069	0.670
Number of children^a^	0.678	0.073	0.219	0.540	0.200
Co‐parenting^a^	0.196	0.660	0.931	0.239	0.851
Living together with the other parent^a^	0.942	0.638	0.915	1.000	0.707
Parent spent childhood in Norway^a^	0.084	0.973	0.845	0.357	0.161
Long‐term disease in parent^a^	0.125	0.275	0.122	0.312	0.031*
Long‐term disease in child^a^	0.134	0.631	0.482	0.499	0.283

	6. Ability to actively engage with healthcare providers	7. Navigating the healthcare system	8. Ability to find good health information	9. Understand health information well enough to know what to do
	*p*	*p*	*p*	*p*
Gender^a^	0.627	0.504	0.311	0.051
Age of parent^a^	0.085	0.229	0.550	0.751
Education level^a^	0.372	0.872	0.316	0.751
Employment status^a^	0.990	0.323	0.299	0.498
Household income^b^	0.548	0.535	0.326	0.538
Age of child^b^	0.091	0.268	0.061	0.127
Number of children^a^	0.706	0.768	0.315	0.379
Co‐parenting^a^	0.617	0.403	0.932	0.912
Living together with the other parent^a^	0.745	0.834	0.874	0.453
Parent spent childhood in Norway^a^	0.987	0.859	0.396	0.921
Long‐term disease in parent^a^	0.155	0.809	0.822	0.614
Long‐term disease in child^a^	0.355	0.706	0.895	0.353

*Note:* With Bonferroni correction of multiple testing, none of the results were significant. Bold values indicates * Significant at *p* < 0.005.

Abbreviation: *p*: sig (2‐tailed).

^a^ Mann–Whitney *U* test.

^b^ Kruskal–Wallis test.
